# Shuttlecock sports integration into physical education to cultivate athletic abilities

**DOI:** 10.3389/fpsyg.2025.1566207

**Published:** 2025-11-06

**Authors:** Yawei Sun, Quanzhi Li, WeiQi Xue

**Affiliations:** 1 Sports Department, Nanjing Agricultural University, Nanjing, China; 2 Sports Training Academy, Nanjing Institute of Physical Education and Sports, Nanjing, China

**Keywords:** shuttlecock sports, physical education, athletic abilities, PE classes, physical activity

## Introduction

In the context of globalization, the general decline in physical activity levels among children and adolescents has become an unignorable public health issue. The World Health Organization (WHO) has recommended a minimum of 60 min of moderate to intense physical activity daily for the 5–17 age group to promote their overall health, including physical health, mental health, and the synchronous development of social skills (Ługowska et al., 2023; Bull et al., 2020). However, the reality falls far short of this ideal. According to numerous international studies, over 80% of adolescents globally do not meet the WHO-recommended levels of physical activity, with this proportion being even more pronounced in China, where only 8.9% of children and adolescents meet the activity standards, well below the global average (Jialin et al., 2017; Lin, 2021; Aubert et al., 2021). The lack of physical activity poses multidimensional threats to adolescent health. Physiologically, a long-term absence of sufficient physical activity not only increases the risk of obesity, cardiovascular diseases, and type 2 diabetes among other chronic non-communicable diseases but can also adversely affect the skeletal system, leading to decreased bone density, osteoporosis, and increased fracture risk (Ługowska et al., 2023; Fukushima et al., 2016). Additionally, the weakening of muscular strength and decline in cardiopulmonary functions are direct consequences of insufficient physical activity, collectively impacting adolescents' overall health (Ługowska et al., 2023). Psychologically, a lack of physical activity is closely associated with issues such as anxiety and depression and can lead to attention deficits and learning disabilities (Fühner et al., 2021). Therefore, exploring effective strategies to enhance physical activity levels in children and adolescents has become a crucial issue needing resolution in the global education and public health sectors.

School Physical Education (PE) programs serve as one of the significant means to promote students' physical activity, and their importance is self-evident. However, current school PE curriculums face numerous challenges during their implementation. The uniformity of the curriculum content, the varying professional abilities of teachers, and the general lack of student interest collectively contribute to a decline in the quantity and intensity of physical activities in PE classes (Cozett and Roman, 2022). Traditional sports, such as track and field and ball games, though beneficial for exercise, often fail to ignite students' enthusiasm due to their lack of novelty and excitement, consequently limiting the enhancement of physical activity levels (Fuentealba-Urra et al., 2022). Thus, how to innovate PE curriculum content and introducing more attractive and challenging sports has become a key pathway to increasing physical activity levels among youths.

Shuttlecock sport, as a simple yet challenging traditional sport, has gradually gained attention from educators and researchers in recent years. With its unique charm, it not only integrates the multi-training effects of bodily coordination, flexibility, and agility but also promotes the development of social skills through team cooperation and interaction. Studies have shown that shuttlecock training can significantly improve the flexibility of children's knee and ankle joints, lumbar and abdominal strength, and cardiopulmonary functions, while effectively enhancing their flexibility and coordination (López Sánchez et al., 2016). These physiological improvements lay a solid foundation for the enhancement of adolescents' overall health. Moreover, as a light-load aerobic exercise, shuttlecock sport plays an active role in promoting cardiopulmonary health and enhancing the adaptability of the cardiovascular system, making it particularly suitable for the adolescent group (Vandoni and Pellino, 2022).

This study aims to explore the feasibility of integrating shuttlecock sports into PE curriculums and their potential impact on students' physical activity (PA) abilities. Based on an analysis of existing research results and the practical effects of shuttlecock activities on students' PA abilities, we discuss the feasibility of incorporating shuttlecock sports into PE curriculums to better facilitate the overall development of students' PA. Through this study, we hope to offer new perspectives and practical guidance for the field of physical education, thus better promoting the development of students' physical health and social adaptability.

## Advantages of shuttlecock sports in physical education programs

With the backdrop of generally declining physical activity levels among adolescents, integrating the traditional and dynamic sport of shuttlecock into physical education courses not only adds new elements to traditional sports education but also showcases its unique advantages in various aspects.

Firstly, the fun and visually appealing nature of shuttlecock sports can capture the interest of students, increasing classroom engagement and student participation fervor. Additionally, shuttlecock combines elements of badminton, volleyball rules, and soccer techniques, offering a net-based, collective sport that not only provides the fitness benefits of traditional sports but also adds elements of opposition and competitiveness, effectively enhancing students' physical fitness (Na, 2020; Lubans et al., 2017; Natal et al., 2023).

Secondly, shuttlecock sports stand out in terms of equipment convenience and cost-effectiveness. Shuttlecocks, as the core equipment, are inexpensive to produce, easy to obtain, and convenient to carry, requiring no complex logistical setup nor costly maintenance, thus significantly reducing the costs of sports education (Dailing, 2014). The reality of limited physical space in many primary and secondary schools poses a challenge (Fang et al., 2023), however, the portability of shuttlecock sports means they are not confined by space, allowing for flexible implementation both indoors and outdoors, making it particularly suitable for schools with limited resources or compact spaces.

Furthermore, shuttlecock sports offer significant advantages in terms of participation flexibility and personalized teaching. Shuttlecock activities can be used as individual practice to improve personal skills or organized into team competitions to strengthen team collaboration. This flexibility not only meets the personalized needs of students at various age levels but also provides sports educators with a variety of teaching strategies to choose from (Gengchen et al., 2023). Teachers can flexibly adjust the content and intensity of the lessons based on the physical fitness level, skill mastery, and personal interests of the students, achieving more precise and effective teaching outcomes.

Research has shown that high school physical education PE teachers who possess a combined model of “professional knowledge + teaching skills” perform better in dimensions such as disciplinary knowledge structure, project-specific skills, and teaching methods. This capability proves to be more effective in organizing shuttlecock-kicking instruction and enhancing students' learning outcomes (Xiaojing, 2024). In terms of pedagogical content knowledge (PCK), experienced PE teachers achieved a significantly higher total score 145.66 compared with novice teachers 107.40, with particularly noticeable differences in the dimensions of student knowledge, instructional objectives, and classroom organization and management. These findings further confirm the decisive influence of teachers' professional competence on teaching quality (Jiaheng, 2017). Furthermore, research grounded in self-determination theory has found that a positive classroom climate and teacher care can stimulate students'intrinsic motivation with enjoyment motivation being the highest, and—through the mediating role of self-efficacy—significantly increase students' willingness to participate in physical activity after class. The path coefficients were 0.65 self–efficacy and 0.47 motivation, respectively (Rui et al., 2024).

Moreover, shuttlecock sports exhibit unique value in skill diversity and comprehensive physical quality improvement. They require participants to have good bodily coordination, balance, reaction speed, and lower body strength. By continually adjusting body posture and controlling the force and direction of shuttlecock kicks, students' fine motor control abilities are trained, while also enhancing cardiopulmonary function and muscle endurance (Na, 2020). With its unique form of exercise, shuttlecock sports provide comprehensive physical training, promoting balanced physical development in students.

## School-based curriculum and practical cases

Overall, Table 1 demonstrates that shuttlecock-kicking covers three educational stages—primary school, junior high school, and high school—indicating that it can play a continuous role throughout students' developmental process. Meanwhile, research has demonstrated its positive effects both in improving students' physical fitness and in fostering their interest in sports.

**Table 1:** School-based curriculum and practical cases.

**Educational stage**	**Pilot schools/regions**	**Curriculum form and implementation highlights**	**Outcomes and experience**
Primary School Rural	Six rural primary schools in Heilongjiang Province	Shuttlecock-kicking incorporated into PE lessons; simple feather shuttlecocks provided; teachers equipped with basic instructional methods via specialized training; conducted 1–2 sessions per week, incorporating gamified teaching	Students' body mass index BMI decreased; physical fitness test results standinglongjump, 50 m sprint improved (Zhuang, 2017).
Primary School Urban	Selected primary schools in Guangzhou City	Under the theme of “Integrating Traditional Sports into Schools,” shuttlecock-kicking interest groups were formed and integrated with after-school club activities	Students' interest in sports increased significantly; after-school participation rate reached 80% (Dongmei, 2019).
Junior High School	Bocai Experimental Middle School, High School Affiliated to Hunan Normal University	“School-based Shuttlecock Curriculum” established, with three modules—techniques, tactics, and competition—integrated with PE lessons and sports clubs	Students progressed from basic entry skills to engaging in group matches; award rate in school sports competitions increased (Zhengyan, 2017).
Higher Vocational Colleges	Multiple higher vocational institutions	Shuttlecock-kicking included as a compulsory component of the “Physical Education and Health” course; adoption of modern educational technologies video demonstrations, motion capture	Students' physical fitness standing long jump, sit–ups improved; classroom satisfaction rate exceeded 90% (Pengxiang, 2019).

## Evidence for integrating shuttlecock sports into physical education

Within classroom design, shuttlecock sports are cleverly integrated into various aspects of the physical education curriculum through group opposition, skill challenges, and team collaboration, creating a fun atmosphere that enhances physical activity abilities. This design not only focuses on the development of students' physical qualities but also fosters team collaboration skills, competitive spirit, and interest in sports.

Shuttlecock sports are a full-body activity that requires participants to constantly move, jump, and pivot, thereby increasing respiratory rate and depth, improving blood circulation, and promoting lung development, effectively enhancing cardiopulmonary function. Studies show significant improvements in pulmonary capacity among boys and girls aged 9–10 after a 12-week shuttlecock intervention in schools (*P* < 0.05), with highly significant differences before and after the experiment (*P* < 0.01) (Xiaobei, 2022). The pulmonary capacity of middle school and high school students also showed significant improvements (*P* < 0.05 and *P* < 0.01, respectively), leading to the conclusion that regular participation in shuttlecock sports significantly enhances adolescents' cardiopulmonary endurance, maintaining higher exercise intensity over extended periods without fatigue (Hanshun, 2012). Additionally, after 18 weeks of shuttlecock training, college students showed significant improvements in maximal oxygen uptake and cardiopulmonary function, indicating substantial benefits to the cardiovascular system from shuttlecock sports (Bo and Zhen, 2017). This enhancement in cardiopulmonary function not only helps adolescents in their daily learning and activities but also lays a solid foundation for engaging in more intensive physical activities in the future (Xiaobei, 2022; Hanshun, 2012).

Shuttlecock sports require participants to react quickly, executing movements such as kicking and catching the shuttlecock in short periods. This rapid, explosive mode of exercise helps improve adolescents' explosive power (Maulida et al., 2024). Studies have demonstrated that adolescents who frequently participate in shuttlecock sports exhibit significantly enhanced explosive power in the legs and waist, enabling them to generate greater strength in short durations—an essential trait for improving performance in sports and daily activities. A systematic 12-week shuttlecock sports program showed significant improvements in boys' and girls' standing long jump and sit-up test scores (*P* < 0.05), although grip strength improvements were not significant (*P* > 0.05). However, improvements in lower body strength and abdominal endurance were more pronounced in the shuttlecock sports teaching group compared to the regular teaching control group, indicating that shuttlecock sports effectively develop lower limb muscles and abdominal strength. There was no significant change in upper limb muscle strength in the control group compared to the shuttlecock group (*P* > 0.05), suggesting that the effect on upper limb muscle strength was not significant (Hanshun, 2012).

Shuttlecock sports significantly enhance adolescents' bodily coordination. For example, the sport requires fast reactions, balance control, and coordination of hands and feet, which not only helps improve flexibility and coordination but also enhances athletic skills (Maulida et al., 2024; Rizal, 2016). Research analyzing the impact of shuttlecock training on the motor coordination abilities of children aged 9–10 showed significant improvements in specific coordination tests (such as the one-leg stand with eyes closed, and single kicks of the shuttlecock) after training, indicating that shuttlecock training strengthens children's neuromuscular systems and enhances external signal processing and nerve excitability (Xiaoliang, 2019). Meanwhile, shuttlecock training effectively exercises the joints, ligaments, and muscles of the lower limbs and waist, and through rapid and continuous motion, promotes coordinated capabilities across various body parts (Fengqiang and Mingye, 2016), significantly improving the flexibility of hip joints and overall balance (Xiaoying, 2021). Additionally, shuttlecock sports emphasize coordination between the eyes and feet, a skill particularly important for mastering shuttlecock techniques (Rizal, 2016).

Shuttlecock sports require participants to maintain high levels of concentration, observing the trajectory of the shuttlecock and the movements of teammates and opponents to make accurate judgments and responses. This continuous concentration training helps enhance adolescents' memory and focus. Studies show that adolescents who frequently participate in shuttlecock sports score higher in memory and concentration tests, indicating that improvements in cognitive abilities help them better focus on their studies and daily lives, enhancing learning efficiency and work productivity (Egger et al., 2019; Rakojević et al., 2016).

## Conclusion

As a simple-to-learn yet challenging traditional sports activity, the integration of shuttlecock sports into the physical education curriculum not only enriches the content but also significantly enhances students' physical activity capabilities. Through shuttlecock training, adolescents show significant improvements in cardiopulmonary function, explosive power, bodily coordination, memory, and focus, covering both physiological and cognitive indicators. Additionally, the convenience and cost-effectiveness of the equipment, along with the flexibility in participation and personalized teaching, make shuttlecock sports a highly suitable physical education option for promotion among the youth. This study confirms the feasibility of integrating shuttlecock sports into physical education programs and their positive effect on enhancing students' overall health levels.

## References

[B1] Aubert S. Brazo-Sayavera J. González S. A. Janssen I. Manyanga T. Oyeyemi A. L. . (2021). Global prevalence of physical activity for children and adolescents; inconsistencies, research gaps, and recommendations: a narrative review. Int. J. Behav. Nutr. Phys. Act. 18, 1–11. doi: 10.1186/s12966-021-01155-2 34187486 PMC8243483

[B2] Bo H. Zhen Z. (2017). The effect of shuttlecock kicking on Cardiovascular Function of College students. J. Zhaoqing Univ. 38, 67–72.

[B3] Bull F. C. Al-Ansari S. S. Biddle S. Borodulin K. Buman M. P. Cardon G. . (2020). World Health Organization 2020 guidelines on physical activity and sedentary behaviour. Br. J. Sports Med. 54, 1451–1462. doi: 10.1136/bjsports-2020-102955 33239350 PMC7719906

[B4] Cozett C. Roman N. V. (2022). Recommendations to enhance parental involvement and adolescent participation in physical activity. Int. J. Environ. Res. Public Health 19:1333. doi: 10.3390/ijerph19031333 35162356 PMC8835355

[B5] Dailing D. (2014). Investigation and Research on Shuttlecock Culture in the Campus of Jinzhong No.1 High School. Inner Mongolia: Inner Mongolia Normal University.

[B6] Dongmei W. (2019). Research on the integration of shuttlecock sports and primary school physical education teaching. Jiangxi Educ. 86.

[B7] Egger F. Benzing V. Conzelmann A. Schmidt M. (2019). Boost your brain, while having a break! The effects of long-term cognitively engaging physical activity breaks on children's executive functions and academic achievement. PLoS ONE 14:e0212482. doi: 10.1371/journal.pone.0212482 30840640 PMC6402646

[B8] Fang Q. Zhang X. Xia Y. Huang F. (2023). Integrating elastic band into physical education classes to enhance strength training. Front. Psychol. 14:1037736. doi: 10.3389/fpsyg.2023.1037736 36874828 PMC9975595

[B9] Fengqiang W. Mingye X. (2016). Feasibility analysis of introducing shuttlecock kicking into the sunshine sports activities for teenagers. Shanxi Agric. Econ. 103. doi: 10.16675/j.cnki.cn14-1065/f.2016.16.078

[B10] Fuentealba-Urra S. Rubio A. Flores-Rivera C. González-Carrasco M. Oyanedel J. C. Castillo-Quezada H. . (2022). Physical activity habits and their relationship with sociodemographic factors in Chilean adolescents. Front. Psychol. 13:915314. doi: 10.3389/fpsyg.2022.915314 36059745 PMC9431025

[B11] Fühner T. Kliegl R. Arntz F. Kriemler S. Granacher U. (2021). An update on secular trends in physical fitness of children and adolescents from 1972 to 2015: a systematic review. Sports Med. 51, 303–320. doi: 10.1007/s40279-020-01373-x 33159655 PMC7846517

[B12] Fukushima N. Inoue S. Hikihara Y. Kikuchi H. Sato H. Tudor-Locke C. . (2016). Pedometer-determined physical activity among youth in the Tokyo Metropolitan area: a cross-sectional study. BMC Public Health 16:1104. doi: 10.1186/s12889-016-3775-5 27769277 PMC5073463

[B13] Gengchen L. Jincheng S. Bingjian Z. (2023). “Research on the development strategies of shuttlecock sports in school physical education in the new era,” in The 13th National Congress of Sports Science (Shanxi Normal University, Chinese Society of Sports Science), 3.

[B14] Hanshun Y. (2012). Experimental Study on the Impact of Shuttlecock Kicking on the Physical Fitness of Middle School Students [D].

[B15] Jiaheng C. (2017). A Comparative Study on Subject Teaching Knowledge (PCK) between Newly Appointed Physical Education Teachers and Experienced Physical Education Teachers. Guangzhou: Guangzhou University

[B16] Jialin Z. Yan T. Peijie C. Yang L. Zhenbo C. Yueying H. . (2017). Research on physical activity of urban children and adolescents in china from a global perspective: a case study of Shanghai. Sports Sci. 37, 14–27. doi: 10.16469/j.css.201701002

[B17] Lin K. (2021). Theoretical and Empirical Research on the Construction of Physical Activity Promotion Models for Chinese Children and Adolescents.

[B18] López Sánchez G. F. González Víllora S. Díaz Suárez A. (2016). Level of habitual physical activity in children and adolescents from the Region of Murcia (Spain). Springerplus 5, 1–6. doi: 10.1186/s40064-016-2033-8 27047712 PMC4816955

[B19] Lubans D. R. Lonsdale C. Cohen K. Eather N. Beauchamp M. R. Morgan P. J. . (2017). Framework for the design and delivery of organized physical activity sessions for children and adolescents: rationale and description of the ‘SAAFE'teaching principles. Int. J. Behav. Nutr. Phys. Act. 14, 1–11. doi: 10.1186/s12966-017-0479-x 28231794 PMC5324233

[B20] Ługowska K. Kolanowski W. Trafialek J. (2023). Increasing physical activity at school improves physical fitness of early adolescents. Int. J. Environ. Res. Public Health 20:2348. doi: 10.3390/ijerph20032348 36767711 PMC9915395

[B21] Maulida M. Ananda T. M. Sadariah S. (2024). Implementasi permainan tradisional engklek guna melatih keseimbangan motorik kasar anak usia dini di tk negeri 004 bunguran timur. Res. Dev. J. Educ. 10, 105–117. doi: 10.30998/rdje.v10i1.20612

[B22] Na H. (2020). Introduction to shuttlecock kicking and the application of basic kicking techniques in actual combat. Stat. Sci. Technol. 38–39.

[B23] Natal Y. R. Bate N. Azi V. M. (2023). Efektivitas model latihan vma-desain sepak kura bagi siswa smp. J. Eduk. Citra Olahraga 3, 127–135. doi: 10.38048/jor.v3i3.2261

[B24] Pengxiang L. (2019). Research on shuttlecock teaching in physical education courses of higher vocational colleges under the new culture dissemination. J. Commun. Power 3:192.

[B25] Rakojević B. Leontijević B. Janković A. (2016). Sensitivity of the speed evaluation tests of carrying the ball in youth soccer players. Fizička kultura 70, 164–171. doi: 10.5937/fizkul1602164R

[B26] Rizal A. (2016). Kontribusi koordinasi mata-kaki dankeseimbangan terhadap keterampilan sepaksila dalam permainan sepaktakraw siswa smpnegeri1 tanasitolo kabupaten wajo. J. Penjakora 3, 1–83.

[B27] Rui Q. Hongyang B. Xu F. (2024). “A study on the influence of students' exercise motivation and sports participation in public physical education classes: the mediating role of self-efficacy,” in The Second Shaanxi Provincial Sports Science Conference (Hubei Normal University), 2.

[B28] Vandoni M. Pellino V. C. (2022). Physical activity and exercise practice to reduce the sedentary behavior in children and adolescents overweight and with obesity. MDPI 2022:5996. doi: 10.3390/ijerph19105996 35627533 PMC9141746

[B29] Xiaobei J. (2022). Research on the Impact of Shuttlecock Training on the Physical Health of Children Aged 9-10. Heilongjiang: Harbin Normal University.

[B30] Xiaojing C. (2024). Research on the enhancement of professional quality of high school physical education teachers by “professional knowledge + teaching skills”. Athletics 73–75.

[B31] Xiaoliang H. (2019). Experimental Study on the Impact of Shuttlecock Training on Motor Coordination Ability of Children Aged 9-10. Shaanxi: Xi'an Physical Education University.

[B32] Xiaoying B. (2021). Experimental Study on the Impact of Shuttlecock Kicking on the Balance Ability of Middle-aged People in Hegang Area. Jilin: Jilin Institute of Physical Education.

[B33] Zhengyan C. (2017). Research and analysis on the implementation of shuttlecock school-based curriculum in junior high schools: a case study of bocai experimental middle school affiliated to hunan normal university. CAI Zhi 192.

[B34] Zhuang G. (2017). Feasibility Study on Introducing Shuttlecock Project into Physical Education Curriculum of Rural Primary Schools in Heilongjiang Province. Heilongjiang: Harbin Sport University

